# Supplementary material for: Materials swelling revealed through automated semantic segmentation of cavities in electron microscopy images
Source: Sci Rep. 2023 Mar 30;13:5178. doi: 10.1038/s41598-023-32454-2 (PMC10063681; doi:10.1038/s41598-023-32454-2)
Supplement: Supplementary file 1 — Supplementary Information. [file 41598_2023_32454_MOESM1_ESM.docx]

**Supplementary Information for**

**Materials Swelling Revealed Through Automated Semantic Segmentation of Cavities in Electron Microscopy Images**

Ryan Jacobs^1^, Priyam Patki^2^, Matthew J. Lynch^2^, Steven Chen^2^, Dane Morgan^1^, Kevin G. Field^2^

^1^Department of Materials Science and Engineering, University of Wisconsin-Madison, Madison, Wisconsin, 53706, USA

^2^Nuclear Engineering and Radiological Sciences, University of Michigan - Ann Arbor, Michigan, 48109 USA

^†^Corresponding author email: rjacobs3@wisc.edu

**SI Note 1: Additional results of model swelling predictions**

Regarding the cavity sizes in **Figure S1A**, we see that the model can predict the average per-image cavity size with high accuracy, with an average (standard deviation) MAE of just 1.02 (0.14) nm, which corresponds to an average (standard deviation) MAPE value of 8.94% (0.84%) error in cavity size. This level of defect size accuracy is consistent with our previous work employing the Mask R-CNN model to detect and quantify dislocation loops and black spot defects in FeCrAl alloys, which showed average (standard deviation) defect percent errors of 7.3% (3.8%) also from random cross validation.[2] While this scale of error can generally be considered small, we show in **Section 2.3** of the main text that the errors in swelling are still mainly due to model errors in the full cavity size distribution, particularly for images containing large (> 15 nm) cavities. Finally, the predictions of per-image cavity density are presented in **Figure S1B**. From the parity plot fit statistics standpoint, our model has the highest errors in predicting cavity density. This is particularly true for images with high cavity densities (> 20 ×10^4^ nm^-2^), where the model has a clear bias to lower values. We show in **Section 2.3** of the main text that the significant errors for images with high cavity densities result from the model having difficulty identifying many small (< 5 nm, or about 2% of the image dimension) cavities. **Table S1** contains a summary of the key classification metrics and materials property metrics for each split, together with the average and standard deviation across all five splits.

**
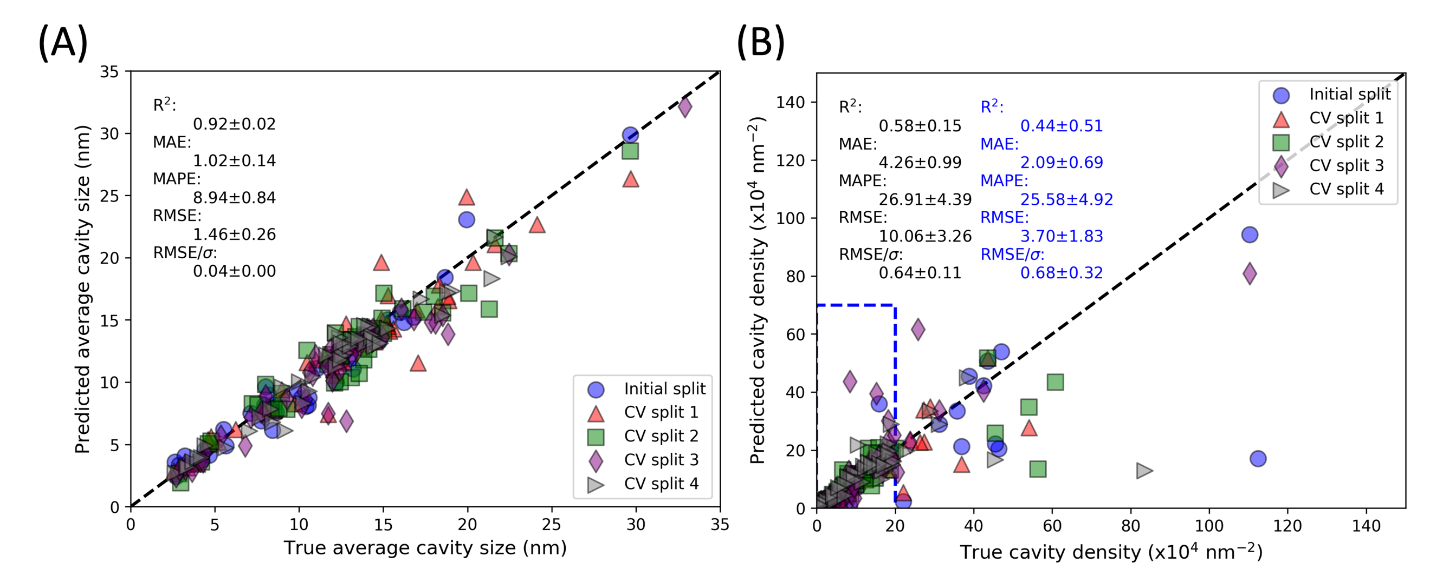
**

**Figure S1:** Parity plot of true and predicted (A) average cavity size, and (B) cavity density. Each data point represents one test image. The different symbols correspond to different cross validation train/test splits. The fit statistics in black text denote the average +/- standard deviation across all five splits for each metric. In (B), the fit statistics in blue text denotes the average +/- standard deviation across all five splits for test images with true cavity density equal or less than 20 x 10^-4^ nm^-2^.

**Table S1:** Summary of classification and material property metrics for five splits of random cross validation using the combined CNL+NOME dataset.

| **Data split** | **Overall statistic** | | | | **Average per-image statistic** | | | **Defect size error (nm) (percent error)** | **Defect density error (x10^4^ nm^-2^) (percent error)** | **Defect swelling error (%) (percent error)** |
| --- | --- | --- | --- | --- | --- | --- | --- | --- | --- | --- |
|  | **P** | **R** | | **F1** | **P** | **R** | **F1** |  |  |  |
| Initial split | 0.74 | 0.62 | | 0.68 | 0.78 | 0.65 | 0.69 | 0.82 (9.73) | 5.94 (25.96) | 0.26 (32.74) |
| CV split 1 | 0.74 | 0.67 | | 0.70 | 0.75 | 0.71 | 0.71 | 1.18 (9.10) | 2.92 (24.00) | 0.35 (20.07) |
| CV split 2 | 0.70 | 0.62 | | 0.66 | 0.73 | 0.71 | 0.70 | 1.10 (9.16) | 3.82 (25.25) | 0.33 (24.78) |
| CV split 3 | 0.72 | 0.62 | | 0.67 | 0.70 | 0.71 | 0.68 | 1.09 (9.38) | 4.53 (35.54) | 0.32 (24.44) |
| CV split 4 | 0.75 | 0.71 | | 0.71 | 0.75 | 0.74 | 0.73 | 0.88 (7.32) | 4.12 (23.80) | 0.27 (19.12) |
| *Average over splits* | *0.73* | *0.65* | *0.69* | | *0.74* | *0.70* | *0.70* | *1.02 (8.94)* | *4.26 (26.91)* | *0.30 (24.23)* |
| *Standard deviation over splits* | *0.02* | *0.04* | *0.02* | | *0.03* | *0.03* | *0.02* | *0.14 (0.84)* | *0.99 (4.39)* | *0.03 (4.82)* |

**SI Note 2: Additional results of leave out group cross validation tests**

Regarding **Figure 3B** in the main text, from the values in **Table S2**, we can see that the model trained and tested on the NOME data performs better on overfocused images than underfocused images, where the swelling MAE values on underfocused (overfocused) images are 0.20 (0.07) percent swelling, respectively. This behavior is opposite to what was observed for the model trained and tested on CNL data. From further inspecting the statistics in **Table S2**, we surmise that the lower swelling error for overfocused images for the NOME model is the result of the lower cavity size errors of just 0.56 nm (vs. 1.02 nm for underfocus), even though the model has worse cavity density errors of 13.84 x10^4^ nm^-2^ for overfocused images (vs. 3.52 x10^4^ nm^-2^ for underfocused images). This result makes sense, given that the swelling scales with the cube of the cavity sizes but only linearly with cavity density (see Eq. 1 in **Section 4** the main text). Therefore, it is more critical to obtain accurate cavity sizes for accurate swelling predictions than accurate cavity densities. This interplay of model errors of cavity size, density and swelling is discussed more in **Section 2.3** of the main text.

**
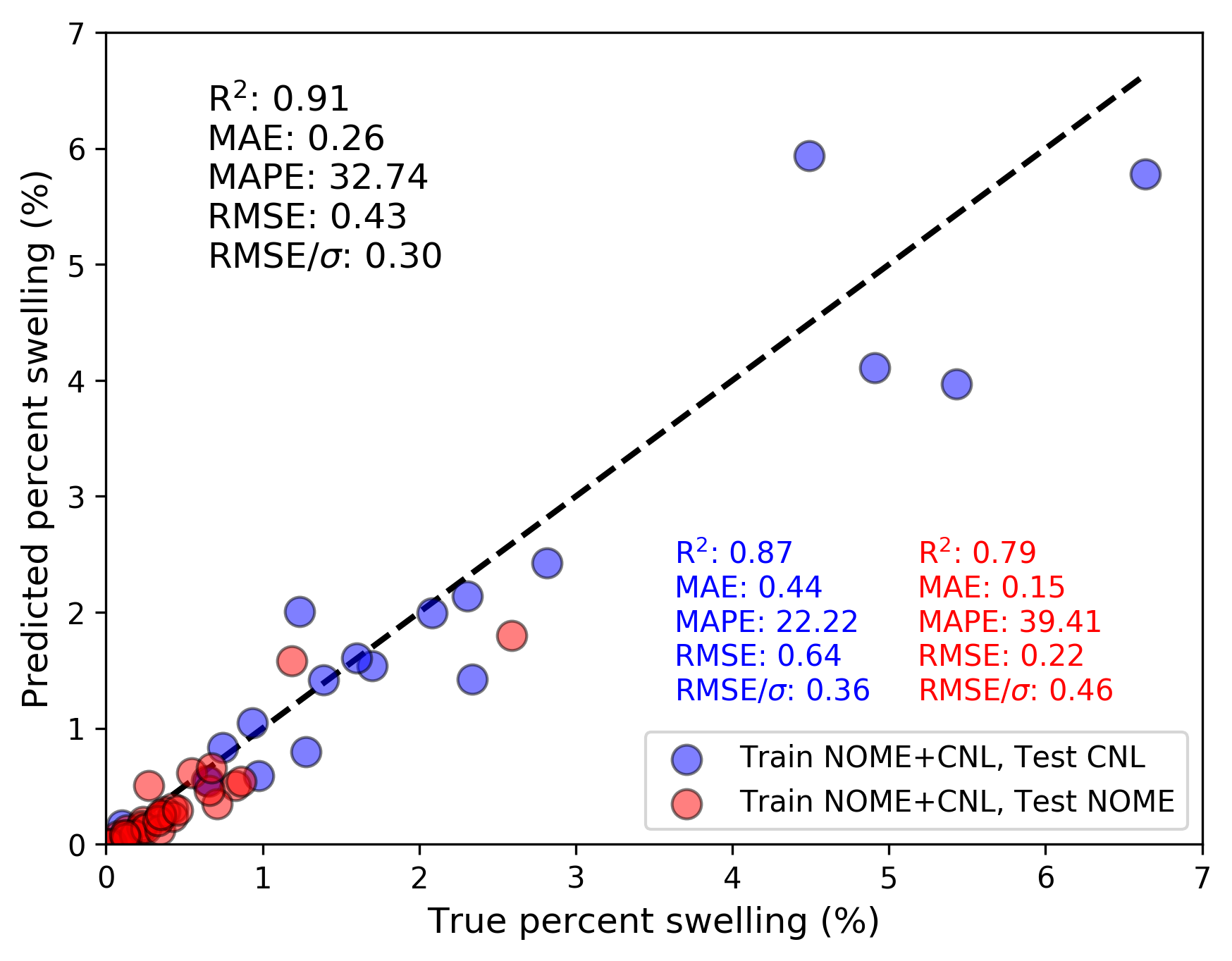
**

**Figure S2:** Additional parity plot assessing Mask R-CNN per-image performance of predicting materials swelling. CNL+NOME initial split, with model trained on CNL+NOME and tested on CNL+NOME, where CNL images are shown as blue points and NOME images as red points.

**Table S2:** Summary of materials property metrics of per-image defect density, defect size, and swelling error for Mask R-CNN models fit to the data splits shown in **Figure 3** of the main text and **Figure S2**. The quoted error values are all MAE values, and the quoted percent errors are all MAPE values.

| **Split name** | **Train** | **Test** | **Figure** | **Defect size error (nm) (percent error)** | **Defect density error (x10^4^ nm^-2^) (percent error)** | **Defect swelling error (%) (percent error)** |
| --- | --- | --- | --- | --- | --- | --- |
| CNL initial split | CNL | CNL | Figure 3A | 0.68 (5.61)  Over: 0.81 (5.87)  Under: 0.60 (5.46) | 2.77 (16.39)  Over: 1.67 (13.83)  Under: 3.41 (17.89) | 0.40 (19.68)  Over: 0.53 (19.08)  Under: 0.33 (20.04) |
| CNL initial split | CNL | NOME | Figure 3A | 4.49 (55.41)  Over: 3.45 (51.43)  Under: 5.08 (57.71) | 14.78 (87.95)  Over: 25.19 (176.0)  Under: 8.75 (36.97) | 0.68 (215.29)  Over: 0.20 (121.28)  Under: 0.96 (269.72) |
| NOME initial split | NOME | NOME | Figure 3B | 0.85 (11.92)  Over: 0.56 (11.90)  Under: 1.02 (11.93) | 7.30 (28.88)  Over: 13.84 (47.79)  Under: 3.52 (17.93) | 0.15 (37.97)  Over: 0.07 (46.22)  Under: 0.20 (33.20) |
| NOME initial split | NOME | CNL | Figure 3B | 6.09 (43.09)  Over: 8.09 (55.59)  Under: 4.93 (35.80) | 12.23 (84.45)  Over: 7.96 (71.84)  Under: 14.73 (91.81) | 1.98 (76.25)  Over: 2.47 (83.08)  Under: 1.70 (72.26) |
| CNL+NOME initial split | CNL+NOME | CNL+NOME | Figure S2 | 0.82 (9.73)  Over: 0.64 (9.00)  Under: 0.93 (10.15) | 5.94 (25.96)  Over: 8.82 (34.38)  Under: 4.27 (21.07) | 0.26 (32.74)  Over: 0.26 (39.13)  Under: 0.27 (29.03) |

Regarding model performance on classification-centric metrics, all models perform better at detecting underfocused cavities than overfocused cavities, where, for example, the CNL+NOME model shows overall (average per-image) F1 scores of 0.72 (0.73) for underfocused images and 0.54 (0.60) for overfocused images, respectively. In addition, when considering underfocused and overfocused images together, the CNL+NOME model shows overall (average per-image) F1 scores of 0.68 (0.69), which are nearly identical to scores of 0.68 (0.73) for the model trained and tested solely on CNL data and to scores of 0.68 (0.66) for the model trained and tested solely on NOME data. Further, we speculate that the model trained solely on NOME data may have a slightly larger domain of applicability than the model trained solely on CNL data. The NOME database contains images of materials from more alloy types and irradiation conditions than the CNL database. From the data in **Table S3**, the model trained on NOME and tested on CNL displays a better overall F1 score of 0.46 than the model trained on CNL and tested on NOME, which has an overall F1 score of 0.39. Overall, the results of **Figure 3** in the main text, **Figure S2** and **Table S3** demonstrate that it is preferable to simply train one model with training images from both datasets, as the model domain is widened without significant loss in classification or materials property metric performance. Finally, it is worth noting that comparisons can be made for the CNL initial split model that is trained and tested on the CNL data with previous findings from the work of Anderson et al.[1] Our model shows overall and average per-image F1 scores of 0.68 and 0.73, respectively, which are lower than the highest F1 score of 0.78 reported in Anderson et al. While it is not clear whether this F1 score of 0.78 reported by Anderson et al. represents an overall or average per-image F1 score, it is nonetheless 0.05-0.1 higher than the F1 scores we obtain here. We attribute this difference to being due to the different number of training images used here compared to Anderson et al. (219 vs. 230 in their work), and the different test set used to evaluate the model performance (19 images vs. 23 images in their work). In addition, different codebases and model types were used between this work and Anderson et al., who used a Tensorflow-based implementation of the Faster R-CNN model, while we use the Mask R-CNN model in Detectron2/Pytorch.

**Table S3:** Summary of classification metrics of per-image P, R, F1 scores and overall P, R and F1 scores for Mask R-CNN models fit to the data splits shown in **Figure 3** of the main text and **Figure S2**.

| **Split name** | **Train** | **Test** | **Figure** | **Overall statistic**  **(Row 1 = All, Row 2 = Overfocus, Row 3 = Underfocus)** | | | **Average per-image statistic (Row 1 = All, Row 2 = Overfocus, Row 3 = Underfocus)** | | |
| --- | --- | --- | --- | --- | --- | --- | --- | --- | --- |
|  |  |  |  | **P** | **R** | **F1** | **P** | **R** | **F1** |
| CNL initial split | CNL | CNL | Figure 3A | 0.72 | 0.64 | 0.68 | 0.76 | 0.72 | 0.73 |
|  |  |  |  | 0.55 | 0.52 | 0.53 | 0.76 | 0.70 | 0.72 |
|  |  |  |  | 0.77 | 0.66 | 0.71 | 0.76 | 0.73 | 0.73 |
| CNL initial split | CNL | NOME | Figure 3A | 0.51 | 0.31 | 0.39 | 0.56 | 0.35 | 0.40 |
|  |  |  |  | 0.30 | 0.18 | 0.22 | 0.49 | 0.23 | 0.29 |
|  |  |  |  | 0.61 | 0.39 | 0.48 | 0.59 | 0.41 | 0.47 |
| NOME initial split | NOME | NOME | Figure 3B | 0.82 | 0.59 | 0.68 | 0.82 | 0.60 | 0.66 |
|  |  |  |  | 0.83 | 0.35 | 0.49 | 0.85 | 0.44 | 0.53 |
|  |  |  |  | 0.82 | 0.72 | 0.77 | 0.81 | 0.70 | 0.74 |
| NOME initial split | NOME | CNL | Figure 3B | 0.62 | 0.36 | 0.46 | 0.47 | 0.23 | 0.26 |
|  |  |  |  | 0.58 | 0.23 | 0.33 | 0.25 | 0.09 | 0.13 |
|  |  |  |  | 0.63 | 0.39 | 0.48 | 0.60 | 0.31 | 0.34 |
| CNL+NOME initial split | CNL+NOME | CNL+NOME | Figure S2 | 0.74 | 0.62 | 0.68 | 0.78 | 0.65 | 0.69 |
|  |  |  |  | 0.65 | 0.47 | 0.54 | 0.78 | 0.56 | 0.60 |
|  |  |  |  | 0.76 | 0.68 | 0.72 | 0.79 | 0.71 | 0.73 |

**SI Note 3: Additional discussion of swelling errors**

**Figure S3** contains a scatter plot of the true per-image average cavity size vs. the true per-image cavity density for all data splits considered. In **Figure S3**, the sizes of the points scale with the true material swelling. It is immediately evident in **Figure S3** that the images with the largest swelling are those with average cavity sizes of about 15 nm and greater, cavity areal densities of about 10/10^4^ =10^-3^ nm^-2^ and higher (note this corresponds to a volume density of about 10^16^ cavities/cm^3^, assuming a thickness of 100 nm), and that the material swelling is much more sensitive to the cavity size than the density, consistent with intuition.


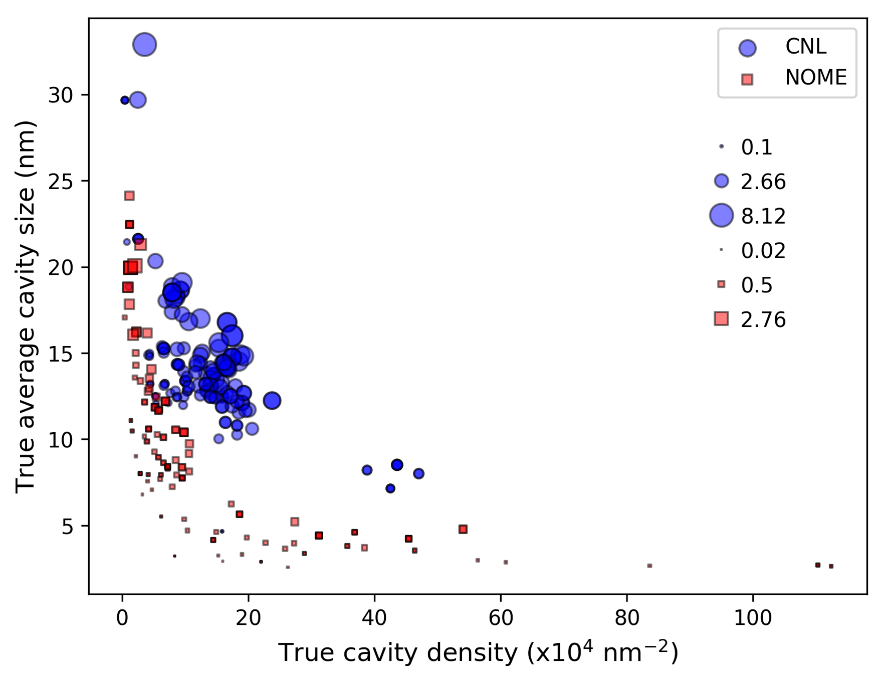


**Figure S3:** Relationship between the true per-image cavity size and the true per-image cavity density. Each data point represents one test image. Respectively, the blue circles and red squares denote CNL and NOME test images. The size of the data points scale with the true percent swelling.


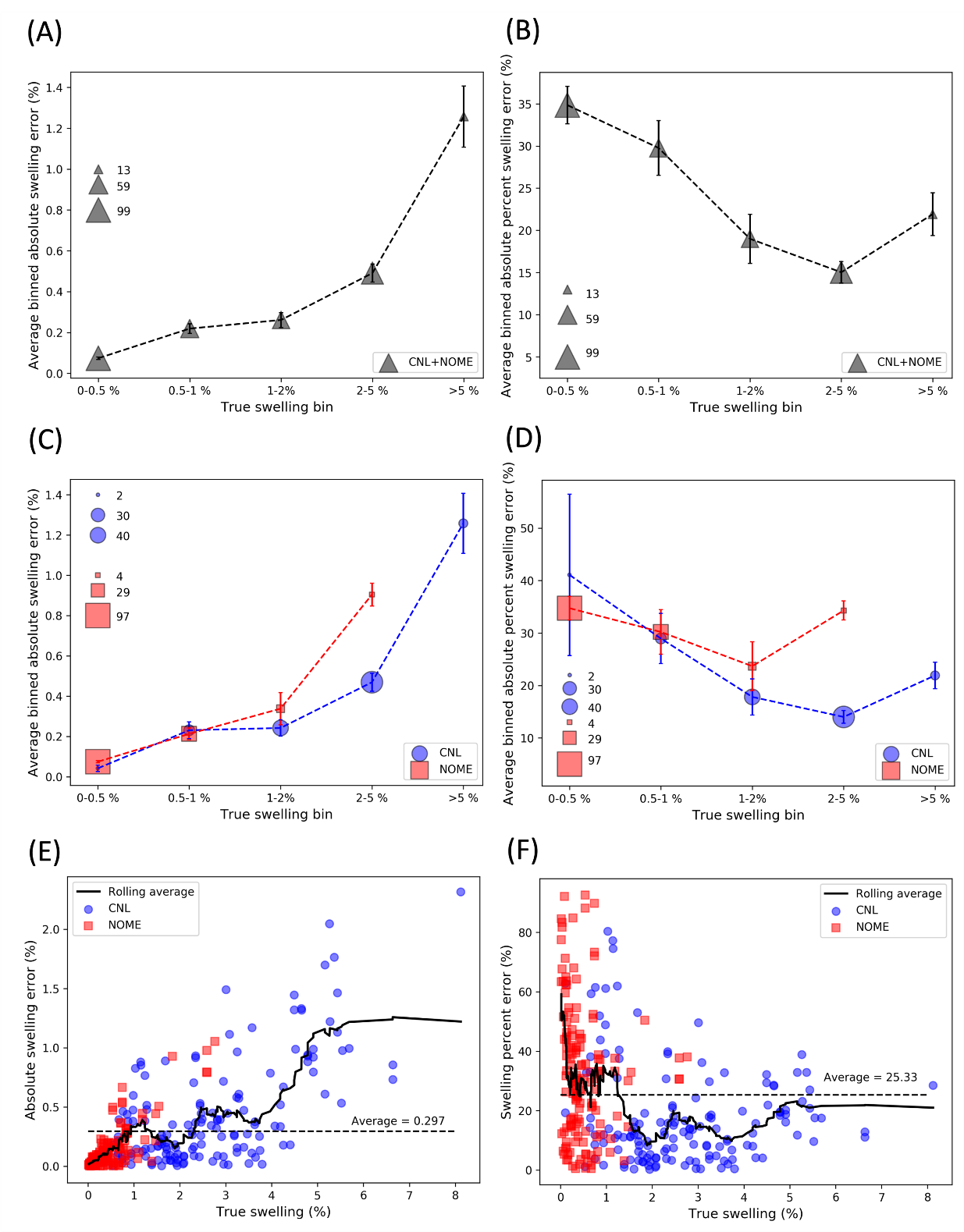


**Figure S4:** (A, C and E) Trend of model absolute error in material swelling as a function of true swelling. (B, D and F) Trend of model absolute percentage error in material swelling as a function of true swelling. In A-D, the x-axis represents binned values of true swelling. In C-F, the blue circles and red squares denote groups of CNL and NOME test images, respectively. The size of the points scales with the number of test images comprising the true swelling bin. The size legends denote the minimum, average, and maximum for the respective data trace. The error bars are the standard error in the mean.

**SI Note 4: Additional information on cavity datasets**

**Table S4:** Summary of image and cavity numbers of the CNL and NOME datasets. Note that some NOME images cannot strictly be classified as over or underfocus, so the total number of NOME images is larger than the sum of over and underfocus images.

| **Dataset** | **Number of images** | **Number of cavities** | **Overfocus images** | **Underfocus images** | **Overfocus cavities** | **Underfocus cavities** |
| --- | --- | --- | --- | --- | --- | --- |
| CNL | 238 | 22,864 | 75 | 163 | 3,826 | 19,038 |
| NOME | 162 | 11,569 | 40 | 107 | 2,651 | 8,300 |

**Table S5** contains information detailing the data used to train and test each model and the total numbers of images and cavities used to train and test each model. In addition, a reference to the respective figure and table containing results of models trained and tested on a given data split are provided. Our data splits consist of models trained individually on the CNL (CNL initial split) or NOME (NOME initial split) datasets, which were then tested separately on the CNL and NOME test sets. The purpose of evaluating the Mask R-CNN model performance on the CNL initial split and NOME initial split was twofold: first, it provided information on model performance for models trained and tested on the same dataset (i.e., train on CNL, test on CNL, and train on NOME, test on NOME), and second, it provided a baseline assessment of the ability to use each model to predict properties of the dataset not used in training (i.e., train on CNL, test on NOME, and train on NOME, test on CNL), providing an assessment of the model applicability domain on test data which is markedly different from the training data in terms of material composition, irradiation condition, and typical cavity size.

After conducting tests of models trained individually on the CNL and NOME datasets, we trained a new model combining the same training and test images used in the CNL initial split and NOME initial split, corresponding to a new model called CNL+NOME initial split. This test aimed to assess whether a model could be developed that effectively extends the applicability domain of cavity detection and quantification to encompass both the CNL and NOME datasets, instead of relying on models trained on separate sub-domains of the data. This combined dataset model was then tested on the same test images of the CNL initial split and NOME initial split, enabling a comparison of the combined model performance to separately predict cavity properties of the CNL and NOME test data.

Finally, we further evaluate the model performance on combined CNL+NOME datasets by random cross-validation of the train and test image sets. The CNL+NOME initial split discussed above is one such split as it had effectively a random group of images pulled out for testing. We constructed an additional 4 random splits (for a total of 5 random splits), which we refer to as CNL+NOME CV split N (N=1-4) in **Table S5** and throughout this work. The purpose of evaluating models with these different random splits of CNL+NOME data was to quantify an expected average and standard deviation in model predictive performance for the scenario where the test images are drawn approximately from the same domain as the training images.

**Table S5:** Summary of data splits used to train and test Mask R-CNN models in this work.

| **Split name** | **Train** | **Test** | **Train images** | **Test images** | **Train cavities** | **Test cavities** | **Figure(s) containing results** | **Table(s) containing results** |
| --- | --- | --- | --- | --- | --- | --- | --- | --- |
| CNL initial split | CNL | CNL | 219 | 19 | 20,082 | 2782 | Figure 3A | Table S2, Table S3 |
| CNL initial split | CNL | NOME | 219 | 30 | 20,082 | 2154 | Figure 3A | Table S2, Table S3 |
| NOME initial split | NOME | NOME | 132 | 30 | 9415 | 2154 | Figure 3B | Table S2, Table S3 |
| NOME initial split | NOME | CNL | 132 | 19 | 9415 | 2782 | Figure 3B | Table S2, Table S33 |
| CNL+NOME initial split | CNL+NOME | CNL+NOME | 351 | 49 | 29,474 | 4936 | Figure 2  Figure 4,  Figure S1, Figure S2, Figure S4 | Table S1, Table S2, Table S3 |
| CNL+NOME CV split 1 | CNL+NOME | CNL+NOME | 350 | 50 | 30,679 | 3756 | Figure 2 | Table S1 |
| CNL+NOME CV split 2 | CNL+NOME | CNL+NOME | 350 | 50 | 30,360 | 4075 | Figure 2 | Table S1 |
| CNL+NOME CV split 3 | CNL+NOME | CNL+NOME | 350 | 50 | 29,765 | 4670 | Figure 2 | Table S1 |
| CNL+NOME CV split 4 | CNL+NOME | CNL+NOME | 350 | 50 | 31,275 | 3160 | Figure 2 | Table S1 |

**SI Note 5: Hyperparameter determination**

The first step to evaluating the performance of our Mask R-CNN models for detecting and quantifying cavities is to choose the value of the IoU threshold and objectness score which maximizes the model performance. **Figure S5** contains two measures of model performance, both of which were obtained by using the CNL+NOME initial split data (see **Table S4** and **Table S5** in **SI Note 4** and **Section 4** of the main text for more information on data splits). **Figure S5A** contains a heatmap plotting the overall F1 score as a function of both IoU threshold and objectness score. From this assessment, we find that an IoU of 0.1 and objectness score of 0.1 result in the best performing model in terms of overall F1 score. **Figure S5B** contains a plot of the mean absolute error (MAE) and root mean squared error (RMSE) of the predicted percent swelling as a function of objectness score. We find that an objectness score of 0.1 results in the lowest swelling MAE of 0.26 percent swelling, and a swelling RMSE of 0.43 percent swelling, slightly higher than the lowest RMSE of 0.42 percent swelling for objectness scores of 0.3 and 0.5. Note that the swelling prediction is only a function of the objectness score and not the IoU threshold. The objectness score determines the total number of predicted cavities per image, thus affecting all of the performance statistics evaluated in this work, while the IoU threshold is used only for classifying when a predicted cavity can be matched with a corresponding true cavity, which in turn affects the P, R and F1 scores only. Based on the findings presented in **Figure S5** of highest overall F1 and lowest swelling MAE for IoU = 0.1 and objectness score = 0.1, for the remainder of this study we evaluate the performance of all models using this set of hyperparameter values. We note that a lower IoU threshold of 0.01 and objectness score of 0.01 resulted in worse performance with an overall F1 score of 0.658, a swelling MAE of 0.27 percent and swelling RMSE of 0.46 percent, indicating the IoU threshold and objectness values of 0.1 and 0.1, respectively, used here likely give optimum model performance, at least on this dataset. In addition, it is worth noting that this optimum IoU value of 0.1 is lower than the IoU value using in our previous work of Mask R-CNN to detect dislocation loops and black dot defects (IoU = 0.3)[2] but comparable to a YOLO model of dislocation loops (IoU = 0.15).[3]


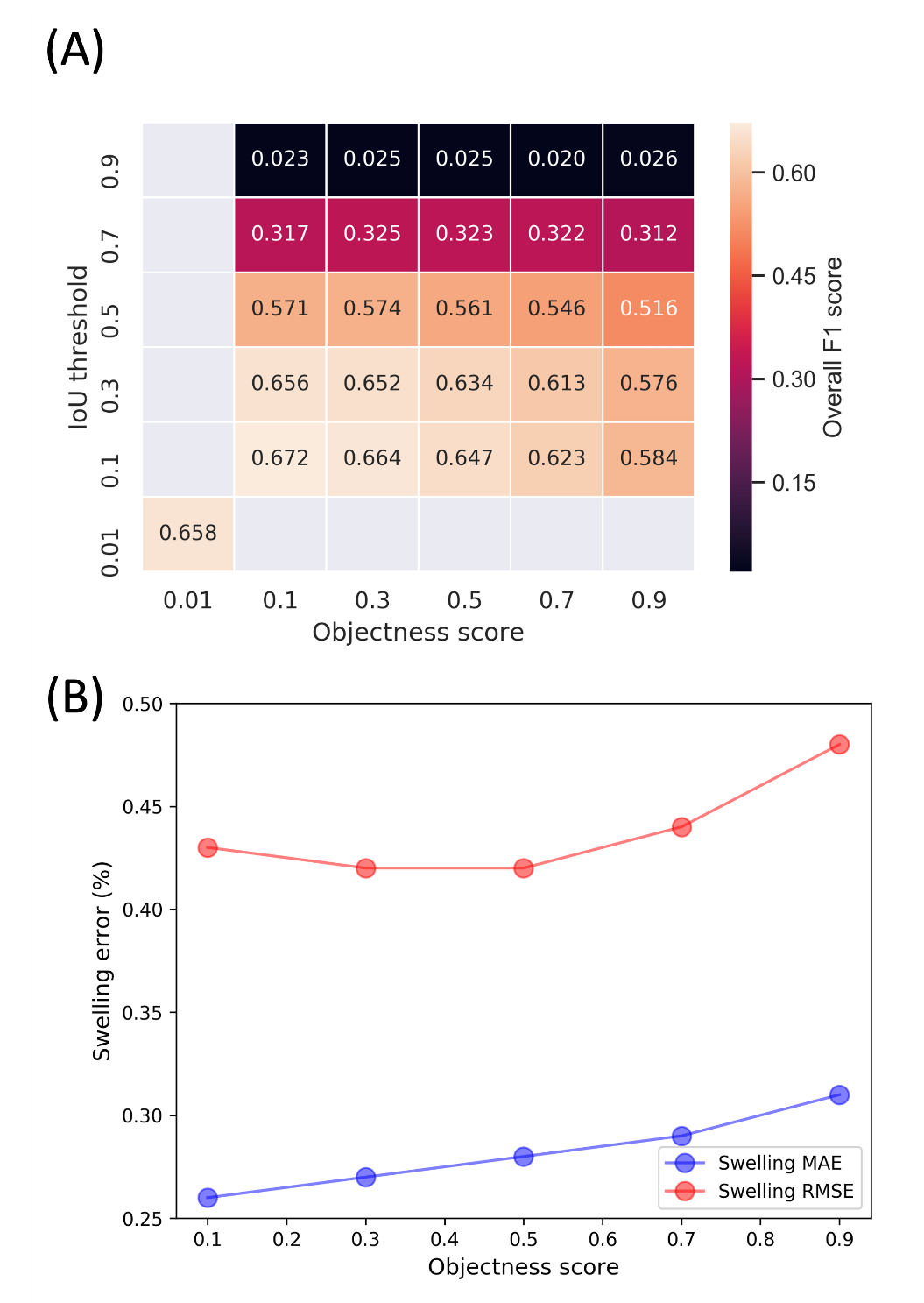


**Figure S5:** (A) Heatmap showing hyperparameter selection to optimize model prediction of material swelling based on the choice of IoU threshold and model objectness score. The heat values correspond to the overall F1 score of the model, where a value of IoU=0.1 and 0.1 objectness score corresponds to the highest overall F1 of 0.672. (B) The mean absolute error (MAE, blue points) and root mean squared error (RMSE, red points) of material swelling as a function of objectness score, with an IoU=0.1. Here, an objectness score of 0.1 results in the lowest swelling MAE of 0.26 percent swelling, and a corresponding RMSE of 0.43 percent swelling. The CNL+NOME initial split was used in these evaluations. Note that lower IoU threshold and model objectness scores of 0.01 resulted in worse performance than the IoU=0.1 and objectness score of 0.1 shown here.

**References**

[1] C.M. Anderson, J. Klein, H. Rajakumar, C.D. Judge, L.K. Béland, Automated Detection of Helium Bubbles in Irradiated X-750, Ultramicroscopy. 217 (2020) 113068. doi:10.1016/j.ultramic.2020.113068.

[2] R. Jacobs, M. Shen, Y. Liu, W. Hao, X. Li, R. He, J.R.C. Greaves, D. Wang, Z. Xie, Z. Huang, C. Wang, K.G. Field, D. Morgan, Performance and limitations of deep learning semantic segmentation of multiple defects in transmission electron micrographs, Cell Reports Phys. Sci. (2022) 100876. doi:10.1016/j.xcrp.2022.100876.

[3] M. Shen, G. Li, D. Wu, Y. Yaguchi, J.C. Haley, K.G. Field, D. Morgan, O. Ridge, O. Ridge, A deep learning based automatic defect analysis framework for In-situ TEM ion irradiations, Comput. Mater. Sci. 197 (2021) 110560. doi:10.1016/j.commatsci.2021.110560.
